# Circular RNAs as emerging regulators in COVID-19 pathogenesis and progression

**DOI:** 10.3389/fimmu.2022.980231

**Published:** 2022-11-09

**Authors:** Xiaojun Gao, Dan Fang, Yu Liang, Xin Deng, Ni Chen, Min Zeng, Mao Luo

**Affiliations:** ^1^ Key Laboratory of Medical Electrophysiology, Ministry of Education, Drug Discovery Research Center, Southwest Medical University, Luzhou, Sichuan, China; ^2^ Laboratory for Cardiovascular Pharmacology, Department of Pharmacology, School of Pharmacy, Southwest Medical University, Luzhou, Sichuan, China; ^3^ College of Integrated Traditional Chinese and Western Medicine, Affiliated Hospital of Traditional Chinese Medicine, Southwest Medical University, Luzhou, Sichuan, China; ^4^ Department of Pharmacy, the Affiliated Hospital of Southwest Medical University, Luzhou, Sichuan, China

**Keywords:** COVID-19, circRNAs, inflammatory response, biological regulator, vaccine

## Abstract

Coronavirus disease 2019 (COVID-19), an infectious acute respiratory disease caused by a newly emerging RNA virus, is a still-growing pandemic that has caused more than 6 million deaths globally and has seriously threatened the lives and health of people across the world. Currently, several drugs have been used in the clinical treatment of COVID-19, such as small molecules, neutralizing antibodies, and monoclonal antibodies. In addition, several vaccines have been used to prevent the spread of the pandemic, such as adenovirus vector vaccines, inactivated vaccines, recombinant subunit vaccines, and nucleic acid vaccines. However, the efficacy of vaccines and the onset of adverse reactions vary among individuals. Accumulating evidence has demonstrated that circular RNAs (circRNAs) are crucial regulators of viral infections and antiviral immune responses and are heavily involved in COVID-19 pathologies. During novel coronavirus infection, circRNAs not only directly affect the transcription process and interfere with viral replication but also indirectly regulate biological processes, including virus-host receptor binding and the immune response. Consequently, understanding the expression and function of circRNAs during severe acute respiratory syndrome coronavirus 2 (SARS-CoV-2) infection will provide novel insights into the development of circRNA-based methods. In this review, we summarize recent progress on the roles and underlying mechanisms of circRNAs that regulate the inflammatory response, viral replication, immune evasion, and cytokines induced by SARS-CoV-2 infection, and thus highlighting the diagnostic and therapeutic challenges in the treatment of COVID-19 and future research directions.

## Graphical Abstract

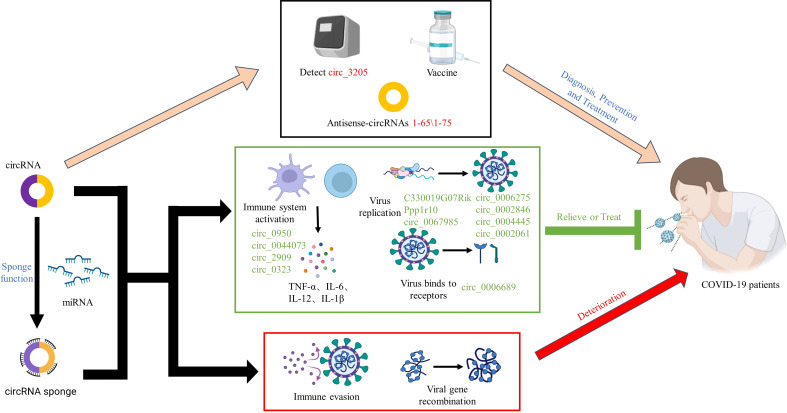

## Introduction

Coronavirus disease 2019 (COVID-19) is an infectious acute respiratory disease caused by severe acute respiratory syndrome coronavirus 2 (SARS-CoV-2) (1). As of 18 July 2022, the cumulative number of cases reported globally is now over 500 million, and the cumulative number of deaths exceeds 6 million. The rapid spread of the provirus strain of SARS-CoV-2 has seriously threatened the lives and health of people across the world, and it certainly caught most of the population completely off-guard and forever changed their lives (2). In times of global pandemic, there is an urgent need for prophylactic vaccines and therapeutic drugs to protect individuals from COVID-19 and to help abate the growing epidemic (1). Currently, several drugs have been used in the clinical treatment of COVID-19, including small molecules (SARS-CoV-2 Mpro inhibitors), neutralizing antibodies (casirivimab and imdevimab), and monoclonal antibodies (tocilizumab) (3–6). In addition, some vaccines have been used to prevent the spread of the pandemic, such as adenovirus vector vaccines (VAXZEVRIA, COVISHIELD™), inactivated vaccines (inactivated COVID-19 vaccine (Vero Cell), CoronaVac), recombinant subunit vaccines (COVOVAX™), and nucleic acid vaccines (COMIRNATY^®^). However, the efficacy of vaccines and the onset of adverse reactions vary among individuals (7). Therefore, it is desperately important to develop safe and effective drugs and vaccines to prevent, diagnose and treat COVID-19.

Circular RNAs (circRNAs) are a large class of abundant, stable and ubiquitous noncoding RNA molecules having a covalently closed loop structure generated from back-splicing of pre-mRNA transcripts. By acting as microRNA (miRNA) sponges, RNA-binding protein sponges, regulating transcription and translating to proteins, circRNAs have recently shown huge capabilities as gene regulators at transcriptional or post-transcriptional levels in the pathogenesis of various diseases, such as viral infections. Accumulating evidence indicates that circRNAs are crucial regulators of viral infections and antiviral immune responses and are heavily involved in COVID-19 pathologies (8–20). During novel coronavirus infection, circRNAs not only directly affect the transcription process and interfere with viral replication but also indirectly regulate biological processes, such as virus-host receptor binding and the immune response (21). However, the characteristics and functional mechanisms of circRNAs in COVID-19 remain unclear. This review focuses on the roles of circRNAs in regulation of the inflammatory response, viral replication, immune evasion, and cytokines induced by SARS-CoV-2 infection, exploring the underlying regulatory mechanisms, and thus highlighting the prospects and challenges in circRNA applications.

## Pathogenesis of SARS-CoV-2 and regulation of related circRNAs

### Structure and pathogenesis of SARS-CoV-2

Coronaviruses (CoVs) are the largest, enveloped, single-stranded positive-sense RNA virus belonging to the Coronaviridae family (22, 23) and have been divided into four genera: α-coronavirus, β-coronavirus, γ-coronavirus, and δ-coronavirus. Additionally, β-coronavirus is further subdivided into four different lineages: A, B, C, and D (24, 25). Genetic sequence analysis has revealed that SARS-CoV-2 and SARS-CoV-1 can be classified into the B lineage, while the Middle East respiratory syndrome coronavirus (MERS-CoV) with lower homology belongs to the C lineage (26, 27). Furthermore, these CoVs possess cis-acting secondary RNA structures flanked by 5’ and 3’ untranslated regions, which are essential for RNA synthesis (28). At the 5’-terminal region, two-thirds of the genomic RNA constitutes two open reading frames (ORF1a and ORF1b), which are involved in encoding nonstructural proteins (nsps) in the viral life cycle. One-third of the genome RNAs of the 3’-end are involved in encoding structural proteins, including spike (S), envelope (E), membrane (M), and nucleocapsid (N) proteins, as well as eight accessory proteins (28–30). Although SARS-CoV-1, MERS-CoV, and SARS-CoV-2 share a large number of similarities in cytopathic effects on host cells, there are fundamental differences in their structures and modes of replication due to sequence divergence. The S protein mediates attachment of the virus to host cell surface receptors and is the first and essential step in CoV infection. However, both SARS-CoV-1 and SARS-CoV-2 enter host cells by using membrane-bound angiotensin-converting enzyme 2 (ACE2) as a primary receptor, while MERS-CoV enters host cells by binding to the dipeptidyl peptidase 4 (DPP4) receptor (31). Furthermore, in addition to relying on cell-surface transmembrane serine protease 2 (TMPRSS2), SARS-CoV-1 entry into host cells also relies on the assistance of cysteine cathepsin B (CatB) and cysteine cathepsin L (CatL), while the invasion of SARS-CoV-2 depends only on TMPRSS2 (32, 33). Moreover, several studies have found that SARS-CoV-1 mainly enters cells by binding to the ACE2 receptor in the lower respiratory tract tissue of the host, whereas SARS-CoV-2 mainly replicates in the upper respiratory tract epithelium (34, 35).

**Figure 1:**
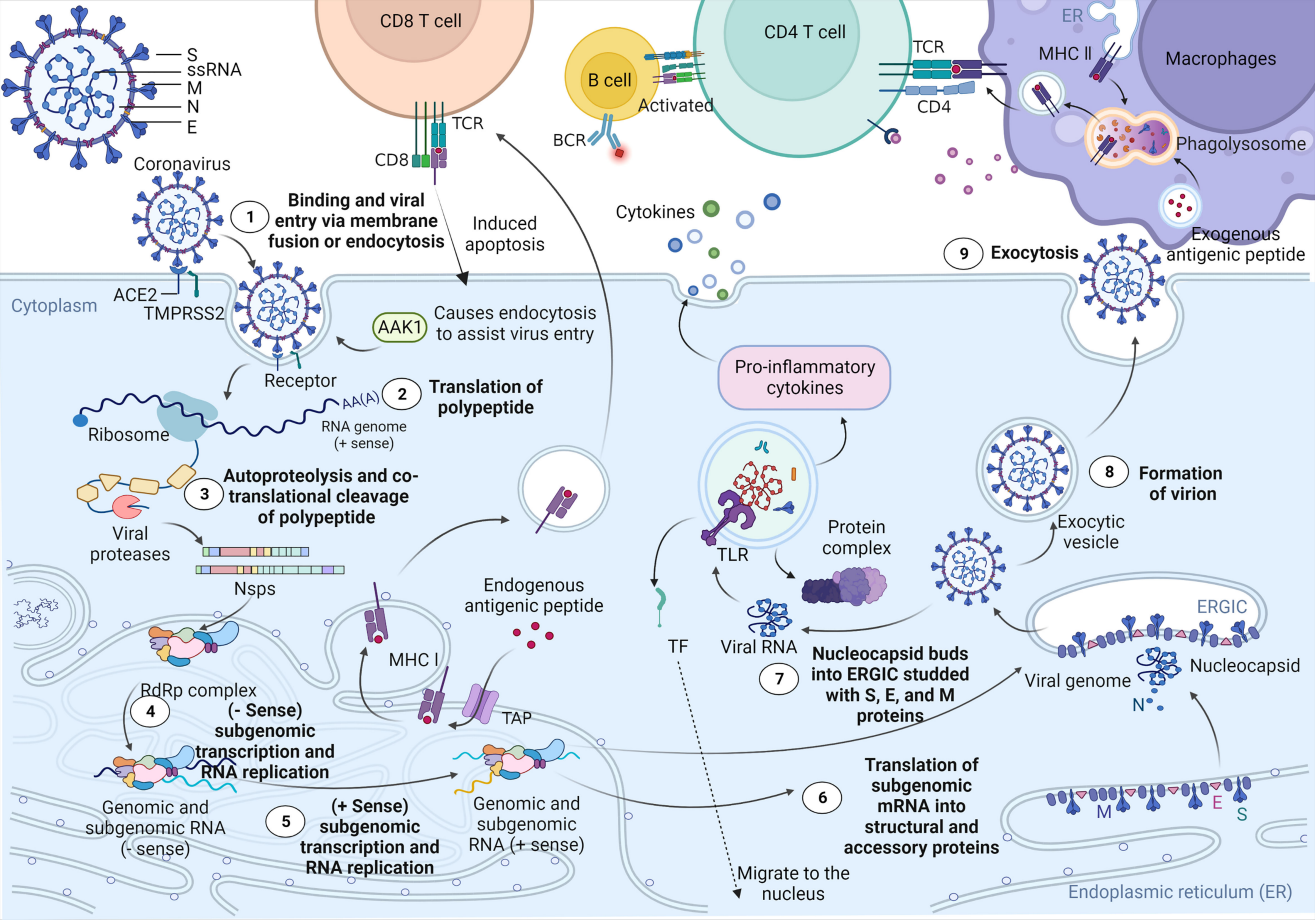
Cell entry mechanism and life cycle of SARS-CoV-2. SARS-CoV-2 virions consist of structural proteins, including spike (S), envelope (E), membrane (M), and nucleocapsid (N) proteins. When in contact with host cells, the S protein of SARS-CoV-2 specifically interacts with cellular receptors [such as angiotensin-converting enzyme 2 (ACE2)] and host factors [such as the cell surface serine protease TMPRSS2 and major endocytosis regulator AP2-related protein kinase 1 (AAK1)] to promote viral uptake and fusion at the cellular or endosomal membranes (37, 38). Following entry, viral genomic RNA is released into the cytoplasm and translated into polypeptides, which are subsequently hydrolysed and cotranslationally cleaved by proteases to generate nonstructural proteins (nsps). nsps further form RNA-dependent RNA polymerase (RdRP) complexes in the endoplasmic reticulum. Subsequently, the RdRP complex is involved in the transcription and RNA replication of the - sense subgenome and the + sense subgenome. Translation of the −sense and +sense subgenomes further enables the synthesis of structural and accessory proteins at the endoplasmic reticulum membrane. At the same time, the nucleocapsid buds into an ER-Golgi intermediate compartment (ERGIC) filled with S, E, and M proteins. Finally, virions are secreted from infected cells *via* exocytosis. As a result, MHC class I contributes towards antiviral immunity by facilitating the presentation of viral antigens to CD8 cytotoxic T cells. Moreover, the ability of antigen-presenting cells to capture external antigens and present them as peptide fragments, loaded on MHC class II molecules, which can transduce signals required for B-cell activation, to CD4+ T cells is a crucial part of the adaptive immune response (39–47). In addition, the RNA released by the virus is captured and recognized by the pattern recognition receptor Toll-like receptor (TLR) located on the endosomal membrane. Subsequently, TLR activates and induces further self-immunity of cells, resulting in the formation of protein complexes, the migration of transcription factors (TFs) to the nucleus, and the expression of proinflammatory cytokines (48, 49).

Recent studies have shown that ciliated bronchial epithelial cells and type II alveolar cells are the primary targets of SARS-CoV-2 (36). As shown in **Figure 1**, when SARS-CoV-2 attaches to host cells, its S1 protein binds to the ACE2 receptor on the surface of the host cells. At the same time, the master regulator of endocytosis, AP2-associated kinase 1 (AAK1), triggers endocytosis for smooth virus entry into host cells. However, loss of AAK1 leads to interruption of virus particle assembly and entry of the virus into susceptible host cells (36). After entering cells, SARS-CoV-2 hijacks the endogenous transcriptional machinery of host cells to replicate and disseminate within the host. First, the RNA of SARS-CoV-2 encodes 2 long polyproteins and 4 structural proteins, of which the polyproteins are hydrolysed by proteases to generate short nsps, which promote viral replication and induce rapid cellular decay (37, 50). The major histocompatibility complex (MHC) class I-mediated antigen presentation is an ubiquitous process by which cells present endogenous proteins to CD8+ T lymphocytes during immune surveillance and response and plays a critical role in antiviral immunity. A recent study by Yoo J et al. showed that the MHC class I pathway is targeted by SARS-CoV-2 (39). Moreover, the induction of the MHC class I pathway is inhibited by SARS-CoV-2 infection. MHC class I contributes towards antiviral immunity by facilitating the presentation of viral antigens to CD8 cytotoxic T cells. Consequently, activated CD8 cytotoxic T cells specifically eliminate virus-infected cells (40–43). In addition, the ability of B lymphocytes to capture external antigens and present them as peptide fragments, loaded on MHC class II molecules, to CD4+ T cells is a crucial part of the adaptive immune response. The ability to activate CD4+ T cells is restricted to antigen-presenting cells that are endocytosed and processed in lysosomes for presentation on MHC class II molecules, which can transduce signals required for B-cell activation. Moreover, MHC class II antigen presentation by B lymphocytes is a multistep process involving in the presentation of MHC II-peptide complexes to CD4+ T cells (44–47). Although the host’s innate immune system works against the virus particles in this process, a small number of viruses still escape, and the RNAs released by these viruses are captured and identified by toll-like receptors (TLRs). Subsequently, activated TLRs further induce cellular autoimmunity, resulting in a series of immune response processes, such as protein complex formation, transcription factor (TF) migration to the nucleus, and proinflammatory cytokine expression (48, 49, 51). Furthermore, several recent studies have demonstrated that SARS-CoV-2 not only directly damages lung tissue but also triggers a cytokine storm, which leads to a sharp increase in cytokines and hyperactivation of immune cells, causing diffuse alveolar damage and exacerbating respiratory failure in patients and even potentially causing uncontrollable systemic inflammatory responses (SIRS) (52).

### Differential expression of related circRNAs

Increasing studies have shown that circRNAs can be used as biomarkers and therapeutic targets in multiple viral diseases, as their abnormal levels may be considered to indicate the stage of pathology and prognosis (53, 54). CircRNAs encoded by viruses might play an important role in host-virus interactions by regulating viral and host gene expression. The study of changes in the expression levels of circRNAs will help us to further understand the mechanism of CoV entry into host cells and how to prevent and treat any secondary symptoms. CircRNAs encoded by CoVs are essential components of the CoV transcriptome and have the potential ability to encode circRNAs exerting different functions in host cells. The dynamic expression of these circRNAs may regulate host gene expression at different times to influence virus pathogenicity. For example, Cai Z et al. (15) identified 28754, 720, and 3437 virus-encoded circRNAs from Calu-3 cells infected with MERS-CoV, SARS-CoV-1, and SARS-CoV-2, respectively. Moreover, the expression levels of MERS-CoV-encoded circRNAs were significantly higher than those encoded by SARS-CoV-1 and SARS-CoV-2 circRNAs. The results revealed that the expression level of certain circRNAs was increased in the late stage of viral infection compared to the early stage. Interestingly, another study came to a different conclusion. Yang S et al. (17) predicted 351, 224, and 2764 circRNAs derived from SARS-CoV-2, SARS-CoV, and Middle East respiratory syndrome coronavirus, respectively. Moreover, 75 potential SARS-CoV-2 circRNAs were identified from RNA samples extracted from SARS-CoV-2-infected Vero E6 cells. These results suggest that virus-encoded circRNAs have strong cell and tissue specificity and play critical roles in autoimmune diseases and viral pathogenesis.

Currently, multiple studies based on bioanalysis have found a variety of dynamically expressed host-encoded circRNAs associated with SARS-CoV-2 infection. Yang M et al. (16) found 42 host-encoded circRNAs that were significantly dysregulated, of which 17 were upregulated and 25 were downregulated in SARS-CoV-2-infected human lung epithelial cells. Dysregulated circRNAs can regulate mRNA stability, immunity, and cell death by binding specific proteins and indirectly regulate gene expression by absorbing their targeted miRNAs. Notably, this result is consistent with that obtained by Zhang X et al. (18) who demonstrated that the proportion of differentially expressed (DE) circRNAs was very low (4/35056, 0.01%) at 6 hpi and was significantly increased (1567/46569, 3.4%) at 24 hpi in MERS-CoV-infected vs. mock-infected Calu-3 cells. Moreover, 1267 DE circRNAs were identified when comparing MERS-CoV-infected samples at 6 and 24 hpi. These results suggest that the DE circRNAs have potential biological functions during CoV infection, especially in the late stage of CoV infection. Additionally, Wu Y et al. (11) identified 570 DE circRNAs, of which 155 were upregulated and 415 were downregulated, in the peripheral blood of COVID-19 patients. Further analysis showed that these circRNAs could negatively affect the normal physiological activities of the body by regulating host cell immunity and inflammation, substance and energy metabolism, cell cycle progression and apoptosis. Collectively, both virus-encoded circRNAs and host-encoded circRNAs are progressively expressed during virus infection, thereby affecting the infection process, but the specific roles and mechanisms remain unclear. As shown in **Supplementary Table 1**, we summarize the key viral and host cell circRNAs and role in COVID-19, SARS and MERS pathogenesis to provide a theoretical basis for further understanding the changes in the expression level of CoV-associated circRNAs and their roles in pathogenesis and virus replication.

## Potential mechanisms of circRNAs in SARS-COV-2 infection

### Sponging of miRNAs by circRNAs affects viral replication

**Figure 2:**
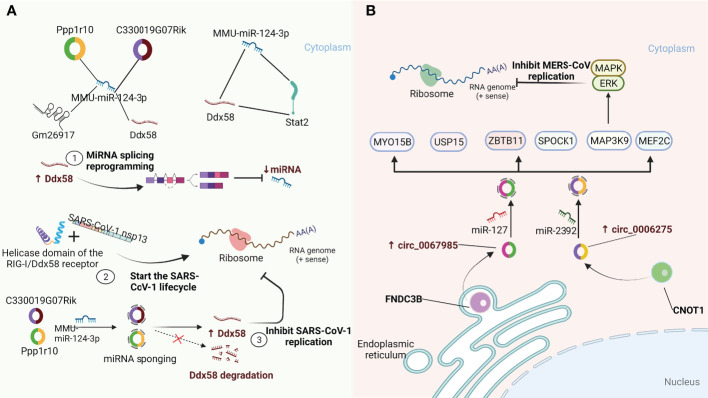
CircRNAs function as miRNA sponges to influence viral replication. **(A)** A quintuple ceRNA network exists in SARS-CoV-1 infection that includes one miRNA (MMU-miR-124-3p), one lncRNA (Gm26917), one TF (Stat2), one mRNA (Ddx58) and two circRNAs (Ppp1r10, C330019G07Rik). They form a closed 3-node miRNA feed-forward loop and a 4-node ceRNA network, respectively. Upregulation of Ddx58 leads to reprogramming of miRNA splicing events, resulting in downregulation of miRNA expression. Meanwhile, the helicase domain of the RIG-I/Ddx58 receptor can interact with SARS-CoV-1 nonstructural protein 13 (NSP13) to initiate the viral life cycle. Furthermore, Ppp1r10 and C330019G07Rik act as sponges for miR-124-3p, inhibiting miR-124-3p expression, which in turn impedes Ddx58 degradation and further inhibits SARS-CoV-1 replication (65). **(B)** circ_0067985 derived from the FNDC3B gene and circ_0006275 derived from the CNOT1 gene serve as miR-127 and miR-2392 sponges, respectively, to regulate the downstream expression of MAP3K9, MYO15B, SPOCK1, MEF2C, USP15 and ZBTB11. Of these, the upstream regulator of the MAPK pathway, MAP3K9, further regulates the downstream ERK/MAPK pathway to inhibit MERS-CoV replication (18).

CircRNAs are highly evolutionarily conserved across species with cell-specific and tissue-specific characteristics (57–60). As an integral part of the competing endogenous RNA (ceRNA) network, circRNAs containing miRNA-responsive elements can regulate downstream target gene expression by acting as microRNA (miRNA) sponges to quickly bind the respective miRNAs and release the inhibitory effect of miRNAs on messenger RNA (mRNA) translation. Remarkably, circRNAs containing miRNA-responsive elements can act as miRNA sponges through the ceRNA network to prevent miRNA-mediated regulation of target genes (61, 62). Moreover, the sponge functions of circRNAs have been confirmed to be more efficient than those of linear miRNAs and long noncoding RNA (lncRNA) transcripts (63, 64). A recent study by Arora S et al. (65) identified a ceRNA network consisting of one miRNA (MMU-miR-124-3p), one lncRNA (Gm26917), one TF (Stat2), one mRNA (Ddx58), and two circRNAs (Ppp1r10, C330019G07Rik) in SARS-CoV-1-infected cells. As shown in **Figure 2A**, the RIG-I/Ddx58 receptor in the ceRNA network has a helicase domain that interacts with SARS-CoV-1 nsp13 and initiates the viral life cycle. In addition, Ddx58 is involved in the mRNA splicing process and miRNA biogenesis, and its upregulation leads to reprogramming of miRNA splicing events, thereby downregulating the miRNA expression. Overexpression of miR-124-3p leads to the degradation of Ddx58, resulting in a reduction in viral replication. Furthermore, miR-124-3p has been shown to modulate TLR-mediated innate immune responses by targeting Stat3 and reducing IL-6 and TNF-α expression (66). These results suggest that miR-124-3p may also play a similar role in regulating Stat2 to affect the viral life cycle. Importantly, in this ceRNA regulatory network, two circRNAs (Ppp1r10 and C330019G07Rik) play vital regulatory roles as sponges of miR-124-3p to hinder the degradation of Ddx58, which in turn affects the replication of SARS-CoV-1. In addition, Zhang X et al. (18) found that host circRNAs mainly function as sponges of miRNAs to affect MERS-CoV replication. As shown in **Figure 2B**, hsa_circ_0067985 is derived from the gene FNDC3B and acts as a sponge of hsa-miR-1275, and hsa_circ_0006275 is derived from the gene CNOT1 and serves as a sponge of hsa-miR-2392, both of which are significantly upregulated in MERS-CoV infection and thus regulate the expression of representative downstream targets, including MAP3K9, MYO15B, SPOCK1, MEF2C, USP15, and ZBTB11. Collectively, these results provide new insights into the regulation of circRNAs and their related signalling pathways as host-targeted antiviral strategies against SARS-CoV-2 infection.

### CircRNAs in regulation of the immune response in SARS-CoV-2 infection by affecting cytokines

CircRNAs can effectively prevent the virus from damaging the body by mediating the immune response process of the host-virus interaction (67). A study by Li X et al. (9) found that NF90/NF110 produced from human interleukin-enhanced binding factor 3 (ILF3) directly regulate back-splicing and coordinate with circRNA production in response to viral infection. The nuclear export of NF90/NF110 upon viral infection contributed in part to a decrease in circRNA production. Moreover, NF90/NF110-circRNP accumulation in the cytoplasm may influence the host immune response. The findings also indicated that circRNAs compete with viral mRNAs for binding to NF90/NF110, and circRNAs may act as a molecular reservoir of NF90/NF110 for a prompt immune response upon viral infection (9, 68). In addition, Chen YG et al. (69) found that circRNAs composed of self-splicing introns can bind to the receptor retinoic acid-inducible gene I (RIG-I) to effectively activate immune signal transduction in the context of viral infection. A recent study also indicated that the overexpressed hsa_circ_0000479 in COVID-19 patients may regulate the expression of IL-6 and RIG-I by sponging hsa-miR-149-5p (55). Additionally, regulation of immune responses by circRNAs generally involves the transduction of signalling pathways and the production of cytokines. A recent comprehensive protein transcription analysis indicated that epidermal growth factor receptor (ErbB), hypoxia-inducible factor-1 (HIF-1), mammalian leukaemia target of rapamycin (mTOR) and tumour necrosis factor (TNF) signalling pathways, among others, were markedly modulated during the course of SARS-CoV-2 infection (70). The parental genes of DE circRNAs enriched in these pathways are associated with numerous antiviral signalling pathways, such as interferon (IFN), chemokines, mitogen-activated protein kinases (MAPKs), and RIG-I-like receptors (58), indicating that circRNAs play regulatory roles in cell signal transduction and immune-inflammatory response during SARS-CoV-2 infection.

**Figure 3:**
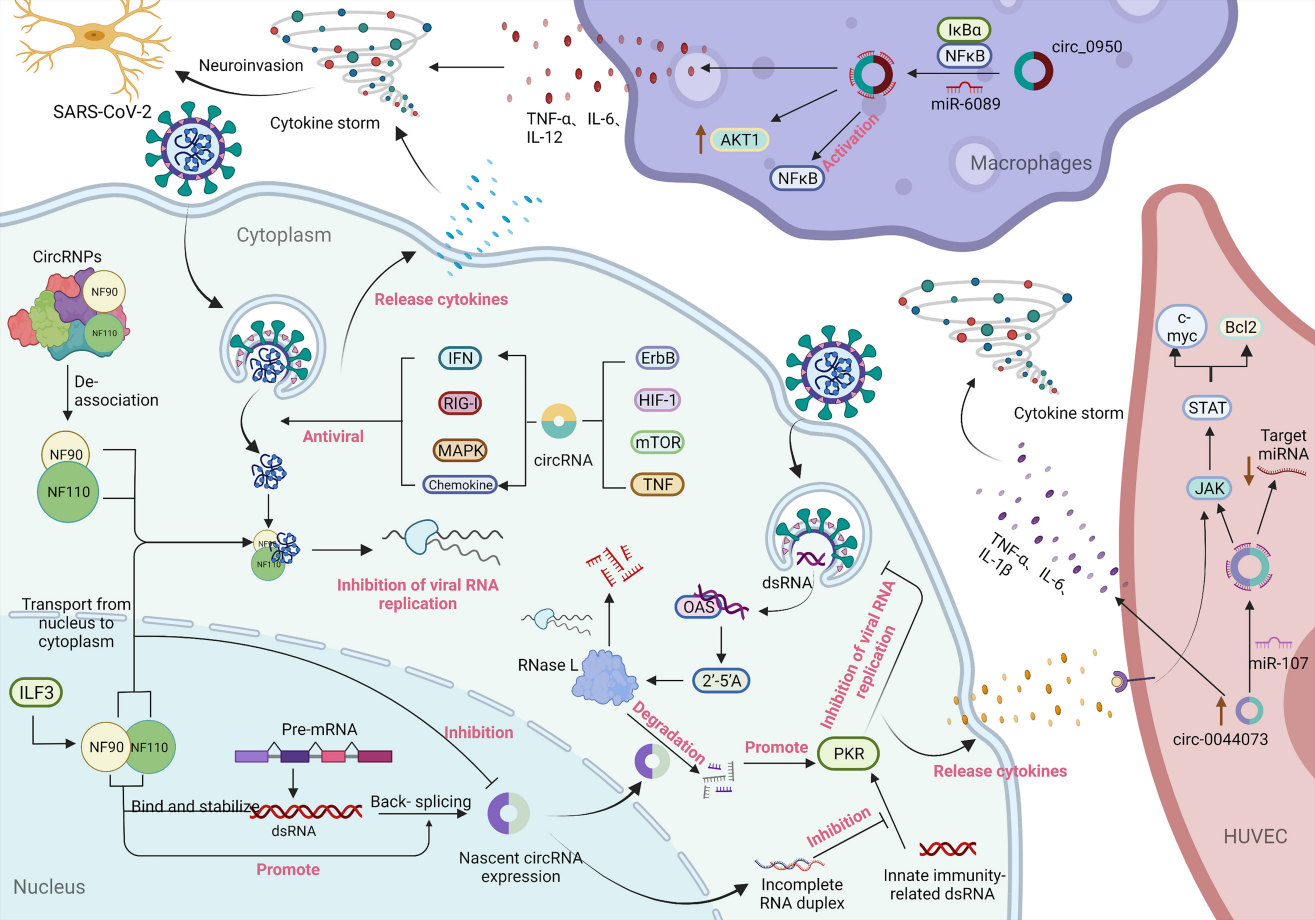
Immune response involving circRNAs in SARS-CoV-2 infection and the potential mechanism of circRNAs in inflammation. During virus infection, nuclear factor 90 (NF90) and its 110 (NF110) isoform produced by interleukin-enhanced binding factor 3 (ILF3) bind to viral mRNA to inhibit virus replication through two pathways: transport from the nucleus to the cytoplasm and decoupling from the circRNA-protein complex (CircRNPs) in the cytoplasm. Among them, the transport of NF90/NF110 from the nucleus to the cytoplasm can reduce the expression of circRNAs. In contrast, the binding of NF90/NF110 to dsRNA formed during pre-mRNA processing can not only stabilize the RNA duplex but also promote reverse splicing to form circRNA (57). 2’-5’ Oligoadenylate (2’-5’A) is generated by the combination of 2’-5’ oligoadenylate synthase (OAS) and viral genome dsRNA and plays an antiviral effect by significantly increasing the activity of RNase L to degrade viral RNA and interfere with viral protein synthesis. Endogenous circRNAs often form incomplete RNA duplexes and act as inhibitors of PKR activation by dsRNAs associated with innate immunity. Meanwhile, circRNAs can be globally degraded by the endonuclease RNase L to activate the PKR antiviral pathway (77). In addition, parental genes enriched for differentially expressed circRNAs in signalling pathways that are significantly regulated upon SARS-CoV-2 infection are associated with multiple antiviral signalling pathways (16, 74, 78). The generation of a cytokine storm is related to the overproduction of proinflammatory cytokines mediated by circRNAs. In macrophages, circ_09505 acts as a sponge of miR-6089 through the IκBα/NFκB signalling pathway, on the one hand, promoting the expression of AKT1 in macrophages and the activation of NF-κB, and on the other hand, promoting the production of the proinflammatory cytokines TNF-α, IL-6 and IL-12 (79). Furthermore, circ_0044073 in HUVSMCs and HUVECs functions as a miR-107 sponge to downregulate the expression levels of target mRNAs, while activation of the JAK/STAT signalling pathway enhanced the expression of the downstream proteins Bcl2 and c-myc. Moreover, circ_0044073 significantly upregulated the levels of the proinflammatory cytokines IL-1β, IL-6 and TNF-α (80). In addition, the occurrence of a cytokine storm disrupts the balance of proinflammatory and anti-inflammatory mechanisms, thereby invading the patient’s nervous system (81, 82).

Cytokines are small molecular polypeptides or glycoproteins synthesized and secreted by a variety of cell types, participating in many physiological processes including the regulation of immune and inflammatory responses. Cytokines have been shown to act as immunomodulators involved in autocrine, paracrine and endocrine signaling, and play important roles in the immune response to host-viral infection (71, 72). Moreover, viral infection can lead to the production of cytokines that have a crucial role in control of the immune response and anti-viral defence, as well as in the capacity of target cells to support virus replication (73). Several recent studies have found that SARS-CoV-2 infection triggers an autoimmune response by activating certain immune factors, such as 2’-5’ oligoadenylate synthase (OAS1-3), interferon-inducible protein (Ifit1-3), and the T helper cell type 1 (Th1) chemokines CXCL9/10/11, and reducing the transcription of ribosomal proteins (74). A previous study showed that the activation of OAS requires the participation of viral genome dsRNA, which combines to generate 2’-5’ oligoadenylate (2’-5’A). Furthermore, 2’-5’A exerts antiviral effects by significantly increasing the activity of RNase L to degrade viral RNA and interfere with viral protein synthesis (75). Liu CX et al. (76) found that endogenous circRNAs tend to form imperfect short (16–26 bp) RNA duplexes and act as inhibitors of dsRNA-activated Protein Kinase R (PKR) associated with innate immunity. Moreover, circRNAs can be globally degraded by the endonuclease RNase L to activate the PKR antiviral pathway. As shown in **Figure 3**, these results indicate that circRNAs may modulate the immune circRNAs may modulate the immune response in SARS-CoV-2 infection by affecting cytokines.

### Potential mechanism of action of circRNAs in inflammation

Inflammation involves a set of biologic mechanisms that evolved in multicellular organisms to contain invasive pathogens and resolve injuries by activating innate and adaptive immune responses, which require a balance between sufficient cytokine production to eliminate pathogens and avoidance of a hyperinflammatory response that causes collateral damage (77). Remarkably, cytokine storm is closely associated with overproduction of a series of proinflammatory cytokines and poor prognosis, which is related to inflammatory signalling in the pathway regulated by circRNAs, as shown in **Figure 3**. A study by Yang J et al. (83) demonstrated that the expression of circ_09505 in arthritic (RA) mice was positively correlated with the life cycle of macrophages. Circ_09505 sponges miR-6089 to promote the expression of AKT1 and activate nuclear factor-kB (NF-κB) in macrophages through the IκBα/NFκB signalling pathway, thereby promoting macrophage inflammation. Furthermore, circ_09505 functions as a sponge of miR-6089 in macrophages to promote the production of proinflammatory cytokines, such as TNF-α, IL-6, and IL-12. Numerous studies have demonstrated that activation of the NF-κB pathway plays a pivotal regulatory role in the development of SARS-CoV-2-induced inflammation (84–88). Collectively, these findings may provide novel insights into the mechanism underlying circRNAs in the regulation of the IκBα/NFκB signalling pathway as a potential therapeutic target for the initial symptoms of inflammation in COVID-19 patients.

Additionally, a study by Shen L et al. (79) found that circ_0044073 was upregulated in chronic inflammation of the arterial vessel wall and promoted the proliferation of human vascular cells by acting as a sponge for miR-107. The study further found that overexpressed circ_0044073 in vascular cells reduced the expression levels of miR-107 target mRNAs and activated the JAK/STAT signalling pathway, thereby enhancing the expression of downstream proteins, such as Bcl2 and c-myc. The JAK/STAT pathway has been shown to be activated downstream of various cytokines during cytokine storms and that is involved in promoting inflammation, proliferation, migration, and adhesion of vascular cells (80). Furthermore, circ_0044073 can significantly increase the levels of proinflammatory cytokines, such as IL-1β, IL-6, and TNF-α. These findings suggested that SARS-CoV-2 disrupts the balance between proinflammatory and anti-inflammatory mechanisms by promoting the occurrence of a cytokine storm *via* the specific regulations of circRNAs pathways, which in turn leads to cardiovascular inflammation in patients with COVID-19.

### Regulatory roles of circRNAs in immune evasion

**Figure 4:**
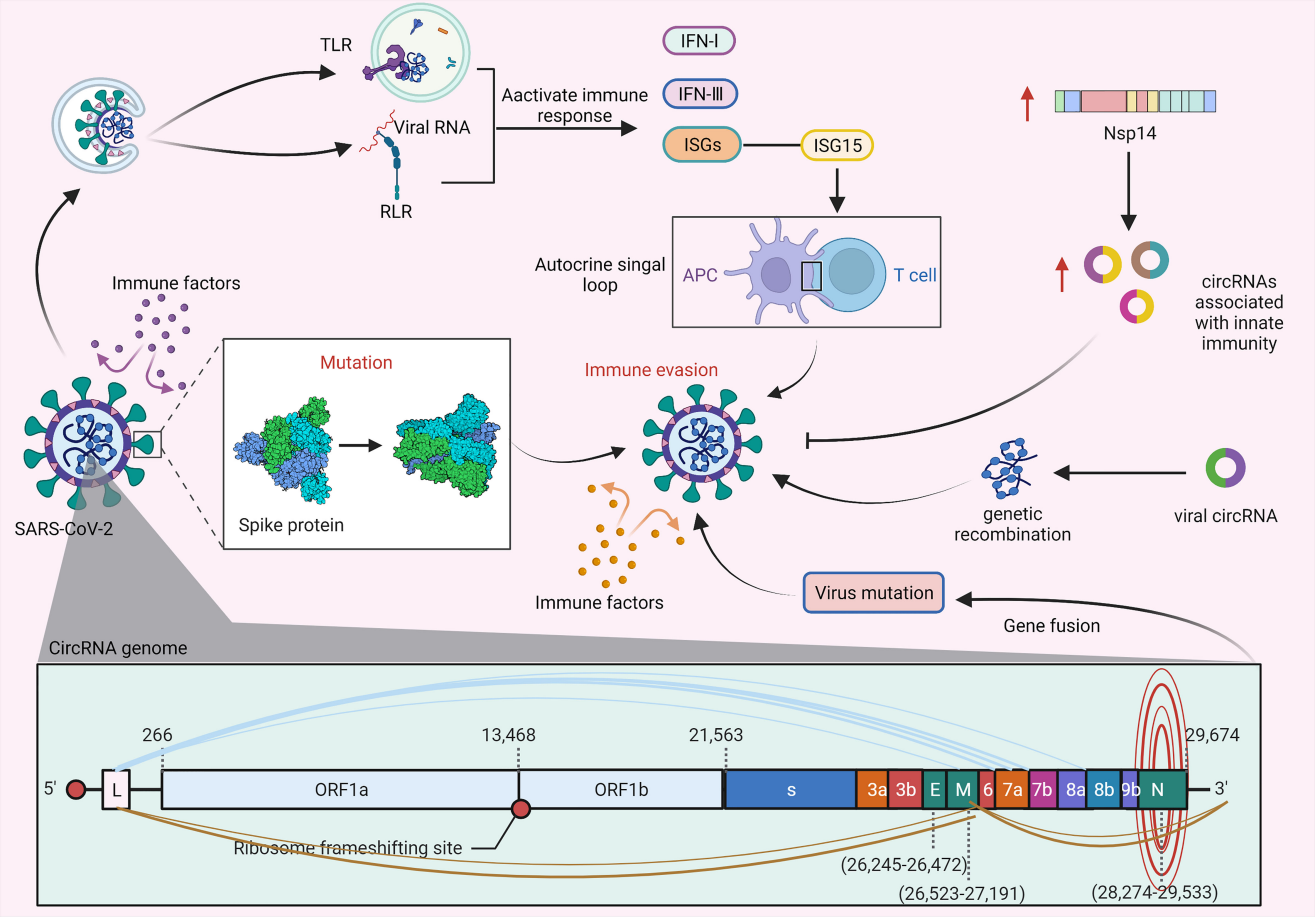
CircRNAs regulate immune evasion of SARS-CoV-2. During SARS-CoV-2 proliferation, genomic RNAs are recognized by TLR receptors and pattern recognition receptors (RLRs) and subsequently activate immune responses. The proviral action of the immune factor ISG15 leads to the generation of an autocrine loop, which amplifies and prolongs autocrine signalling and ultimately induces viral drug resistance (89, 90). Furthermore, the S protein of SARS-CoV-2 can prevent the virus from being cleared by immune factors, and mutation of the S protein will further enhance the ability of the virus to evade immune responses (91, 92). In addition, the expression of viral nsp14 can upregulate the levels of circRNAs related to innate immunity, thereby inhibiting viral replication and immune evasion (93). Likewise, viral circRNAs may be involved in the mechanism of genome recombination, resulting in mutation of virions leading to immune evasion. There are various types of gene fusions in the circRNA genome of SARS-CoV-2, which may cause the virus to mutate and evade immunity.

The innate immune response can effectively prevent the virus from invading the host. However, the virus has evolved the ability to evade the host immune response. As shown in **Figure 4**, viral nucleic acid intermediates and released genomic RNAs during the proliferation of SARS-CoV-2 can be recognized by TLRs and RIG-I-like receptors (RLRs), thereby activating the body’s immune pathways to produce immune factors, such as IFN-I, IFN-III, and numerous ISGs. ISGs have general antiviral effects, however, the proviral effects of ISG15 may lead to the generation of autocrine loops that ultimately induce viral drug resistance by amplifying and prolonging their secretion (89, 94). In addition, a study by Arora S et al. (65) demonstrated that SARS-CoV-1 may reprogram splicing events by inducing the cytoplasmic translocation of DROSHA (an enzyme involved in miRNA biogenesis) to generate another circRNA that can act as a sponge for miR-124-3p and hinder its degradation of Ddx58, thereby evading ISG-mediated antiviral effects (90). Furthermore, SARS-CoV-1 can enhance viral replication by confiscating the helicase in Ddx58 independent of interferon-related pathways.

Several recent studies have found that SARS-CoV-2 can further evade immune responses by mutating its S protein on the basis of its S protein shielding immune factor clearance, which can greatly promote the spread of SARS-CoV-2 in the population (91, 95). Recent studies have shown that circRNAs play a key role in the immune evasion and replication of SARS-CoV-2. For example, Zaffagni M et al. (92, 96) found that SARS-CoV-2 Nsp14 mediates the effects of viral infection on the host cell transcriptome. Nsp14 altered the splicing of more than 1000 genes and resulted in a dramatic increase in the number of circRNAs that were linked to innate immunity. Furthermore, a recent study by Hassanin A et al. (97) indicated that viral circRNAs are involved in the mechanism of genome recombination, which may cause virion mutation leading to immune evasion. In addition, a study by Yang S et al. (17, 98) found that abundant and diverse circRNAs are generated by SARS-CoV-2, SARS-CoV and MERS-CoV and represent a novel type of circRNA that differs from circRNAs encoded by DNA genomes, and forward splice junctions (FSJs) representing noncanonical “splicing” events were detected in these circRNA genome sequences. Furthermore, the study also reported the existence of alternative back-splicing events in SARS-CoV-2 circRNAs that share either 5’ or 3’ breakpoints. Several studies have reported that gene fusion is closely related to the occurrence and development of various diseases, and thus, fused genes may be potential drug targets. Accordingly, atypical fusions in the SARS-CoV-2 transcriptome may provide conditions for the generation of viral mutation as well as viral survival and immune evasion in infected tissues.

## Prospects of the clinical application of circRNAs in COVID-19

### CircRNAs as diagnostic biomarkers for COVID-19 detection

CircRNAs are resistant to RNase R degradation and have a longer half-life, and their expression patterns are affected by viral infection. Several studies have demonstrated that circRNAs are abundant in the circulatory system of COVID-19 patients and may be reliable biomarkers of disease progression or prognosis (15–17). A study by Wu Y et al. (11) found that 114 DE circRNAs in SARS-CoV-2-infected peripheral blood were associated with exosomes, which could not only promote infection but also activate the body’s immune response (99, 100). Moreover, recent studies have demonstrated that exosomes may be a key factor in the recurrence of COVID-19 (101). Importantly, since exosomes can reflect the pathological state of the cells from which they originate, they can be used as diagnostic markers of various diseases. Thus, identification and isolation of exosome-related circRNAs may be helpful for the diagnosis of COVID-19.

In addition, a recent study found that virus-encoded circRNAs in SARS-CoV-2 infection downregulated genes related to cholesterol, alcohol, sterol, and fatty acid metabolic processes and upregulated genes associated with cellular responses to oxidative stress at the later stage of virus infection (15). Barbagallo D et al. (56) found that circ_3205 encoded by SARS-CoV-2 serves as a sponge of hsa-miR-298, thereby targeting downstream KCNMB4 and PRKCE mRNAs to promote the development of COVID-19. Furthermore, circ_3205 was only expressed in positive samples, and its expression was positively correlated with S protein mRNA and SARS-CoV-2 viral load, suggesting that circ_3205 could be used as a diagnostic marker for COVID-19. More importantly, the dysregulation of circRNAs may reflect physiological and pathological changes in each human body. For example, host circRNAs formed by nonsequential back-splicing in SARS-CoV-2-infected Calu-3 cells are widely and abundantly expressed in human lung epithelial cells compared to normal Calu-3 cells (16, 102). Zhang X et al. (18) found that differential expression of circRNAs in the circRNA-miRNA–mRNA network leads to the disturbance of a series of biological processes in MERS-CoV-infected Calu-3 and HFL cells. Overall, it is suggested that monitoring the expression level of circRNAs may provide a new reference index for diagnosis and prognosis determination in COVID-19 patients (103).

### CircRNAs as potential therapeutic targets for COVID-19

Since the outbreak of COVID-19, a wealth of studies have shown that angiotensin-converting enzyme 2 (ACE2) is a recognized receptor for SARS-CoV-2 entry into host cells. Therefore, regulating the ACE2 gene promoter to interfere with the transcription and production of ACE2 may be a new approach to preventing the virus from binding to host cells to prevent COVID-19. Previous studies have shown that sex-determining region Y (SRY) can inhibit ACE2 promoter activity, thus increasing angiotensinogen, renin, and ACE gene promoter activity (104). Furthermore, a recent study by Wang D et al. (105) indicated that there are 24 common transcription factor binding sites in the conserved region of the ACE2 gene promoter, including SRY, HNF-1, IRF, AP-1, YY1, and c-Jun. Previous studies have revealed that SRY transcripts mainly exist in the form of circRNA molecules, which account for more than 90% of all SRY transcripts (106, 107). Moreover, recent studies have also demonstrated that circRNAs can participate in the regulation of SRY-box transcription factors through the ceRNA network thereby regulating the expression of downstream target genes (108–110). Remarkably, several studies have found significant gender differences in the incidence of COVID-19, with males having significantly higher rates than females. Consequently, circRNAs may affect the transcription process of ACE2 by regulating SRY-related genes, thus mediating the infection of SARS-CoV-2 to the host. However, no research has focused on circRNAs involved in the regulation of ACE2 through modulation of the SRY gene. In addition, recent studies have revealed that AXL receptor tyrosine kinase (AXL), a founding member of the TAM family of receptor tyrosine kinases (RTKs), is a novel candidate receptor for SARS-CoV-2 to invade host cells. AXL can specifically interact with the N-terminal domain of the SARS-CoV-2 genome to mediate its entry into host cells without the involvement of ACE2, suggesting that AXL may be a potential target for future treatment of COVID-19 (111). Notably, a recent study has found that hsa_circ_0006689 regulates the transmembrane receptor protein tyrosine kinase signal transduction pathway by targeting hsa-miR-1255a (112), indicating that hsa_circ_0006689 may participate in the invasion of SARS-CoV-2 by mediating the expression of AXL. Taken together, circRNAs may be therapeutic targets against COVID-19 by regulating the binding of SARS-CoV-2 to relevant host receptors.

Immunity induced by a viral infection can protect cellular functions, resist viral invasion, clear viruses, and clear infections. However, excessive activation of immune responses may cause serious damage to the host (113). For example, over-recruitment of immune cells and uncontrolled proinflammatory cytokines can lead to systemic inflammation that can cause extensive damage to tissues and organs. Numerous studies have shown that TNF and IL-1β can stimulate the production of IL-6, which can serve as a biomarker of disease severity and a prognostic indicator of cytokine storm (114, 115). Interestingly, IL-6 is involved in the activation of the NF-kB pathway, and subsequently, NF-kB positively regulates HIF-1α, which in turn enhances the regulatory effect of HIF-1α on the expression of downstream proinflammatory factors. Meanwhile, HIF-1α plays a key role in the synthesis of IL-1β (116–120). A recent study by Tian M et al. (121) found that the ORF3a protein of SARS-CoV-2 in patients with COVID-19 can promote the production of HIF-1α, which regulates the expression of inflammatory cytokines, such as IFN-β, IL-6, and IL-1β, to promote virus replication and infection. Noticeably, Yang YW et al. (122) found that two circRNAs (circ_2909 and circ_0323) could promote the expression of HIF-1α and inducible nitric oxide synthase (NOS2) by acting as sponges for miRNAs. Furthermore, Demirci YM et al. (123) reported DE miRNAs during SARS-CoV-2 infection and found that ORF3a protein is a viral target of human miRNAs. The study further found that among 2498 miRNAs with predicted targets, 2448 had more targets in circRNAs. Therefore, regulatory network of circRNA-miRNA-mRNA contributes to regulating the expression of downstream HIF-1 pathway genes and that may become a new approach to treating COVID-19.

Currently, CoVs have evolved to the point where they can evade a complex system of sensors and signalling molecules to suppress host immunity. Papain-like protease (PLpro) is an enzyme in CoVs that regulates viral spread and innate immune responses (124, 125). Several recent studies have demonstrated that SARS-CoV-2-PLpro is a multifunctional enzyme with deubiquitination and de-ISG activities *via* regulating multiple signalling pathways, such as STING, NF-κB, and TGF-β, to block immune responses (126), suggesting that PLpro can serve as an important therapeutic target against COVID-19. Remarkably, previous studies have indicated that MERS-CoV-PLpro has deubiquitination activity and participates in the proteolysis of viral polyproteins during viral replication (127, 128). A study by Zhang X et al. (18) found that ubiquitin-mediated proteolysis was significantly disturbed after MERS-CoV infection, and DE circRNAs related to ubiquitin-mediated proteolysis could affect MERS-CoV replication by regulating downstream target genes. For example, knockout of hsa_circ_0067985 or hsa_circ_0006275 significantly reduced the expression of MAP3K9, thereby regulating the extracellular signal-regulated kinase (ERK)/MAPK pathway associated with MERS-CoV replication (129). In addition, heterogeneous nuclear ribonucleoprotein C (hnRNP C) is an upstream regulator of multiple proviral circRNAs and can bind and obscure Alu on pre-mRNA and protect against Alu exonation to regulate circRNA biogenesis (130, 131). A recent study by Zhang X et al. (19) found that hnRNP C was able to regulate MERS-CoV replication by targeting the CRK-mTOR signalling pathway. Furthermore, this study also confirmed that hnRNP C is a key modulator of the expression of MERS-CoV-perturbed circRNAs, such as hsa_circ_0002846, hsa_circ_0002061 and hsa_circ_0004445, and the data further demonstrated that hnRNP C regulates the expression of these circRNAs through direct physical binding. The correlation analysis of circRNAs and their parental genes as potential biomarkers and therapeutic targets for the diagnosis of SARS-CoV-2 is summarized in **Supplementary Table 2**, which suggests that circRNAs play a key role in the occurrence of COVID-19.

## Discussion

In summary, circRNAs are emerging as important players in regulating virus-mediated infection and subsequent disease status. With the rapid development of high-throughput sequencing technology and bioinformatics, it has been demonstrated that a large number of circRNAs are DE in COVID-19 patients and that circRNAs play a key role in the process of virus-host interaction. On the one hand, the host directly regulates immune response factors during virus infection *via* circRNAs and indirectly regulates the expression of downstream target genes through the ceRNA network to inhibit virus replication. On the other hand, viruses trigger molecular expression through host-encoded and self-encoded circRNAs, generating new circRNAs that interfere with the host’s innate immune response and that may create a suitable microenvironment for the virus to replicate or mutate in cells. Moreover, conserved circRNAs are widely involved in cell proliferation, differentiation, and apoptosis. Furthermore, these circRNAs act as key targeted therapies for SARS-CoV-2-infected cells, including blocking the binding of the virus to host receptors, inducing host-specific immune responses, interfering with gene transcription, and hindering protein translation. Current studies clearly show that circRNAs are evolutionarily conserved and closely involved in the process of SARS-CoV-2 infection, which paves the way for further studies on how circRNAs regulate host-virus dynamics in CoV-involved diseases. However, the lack of research on clinical effectiveness has greatly increased the challenges in the clinical application of circRNAs.

**Figure 5:**
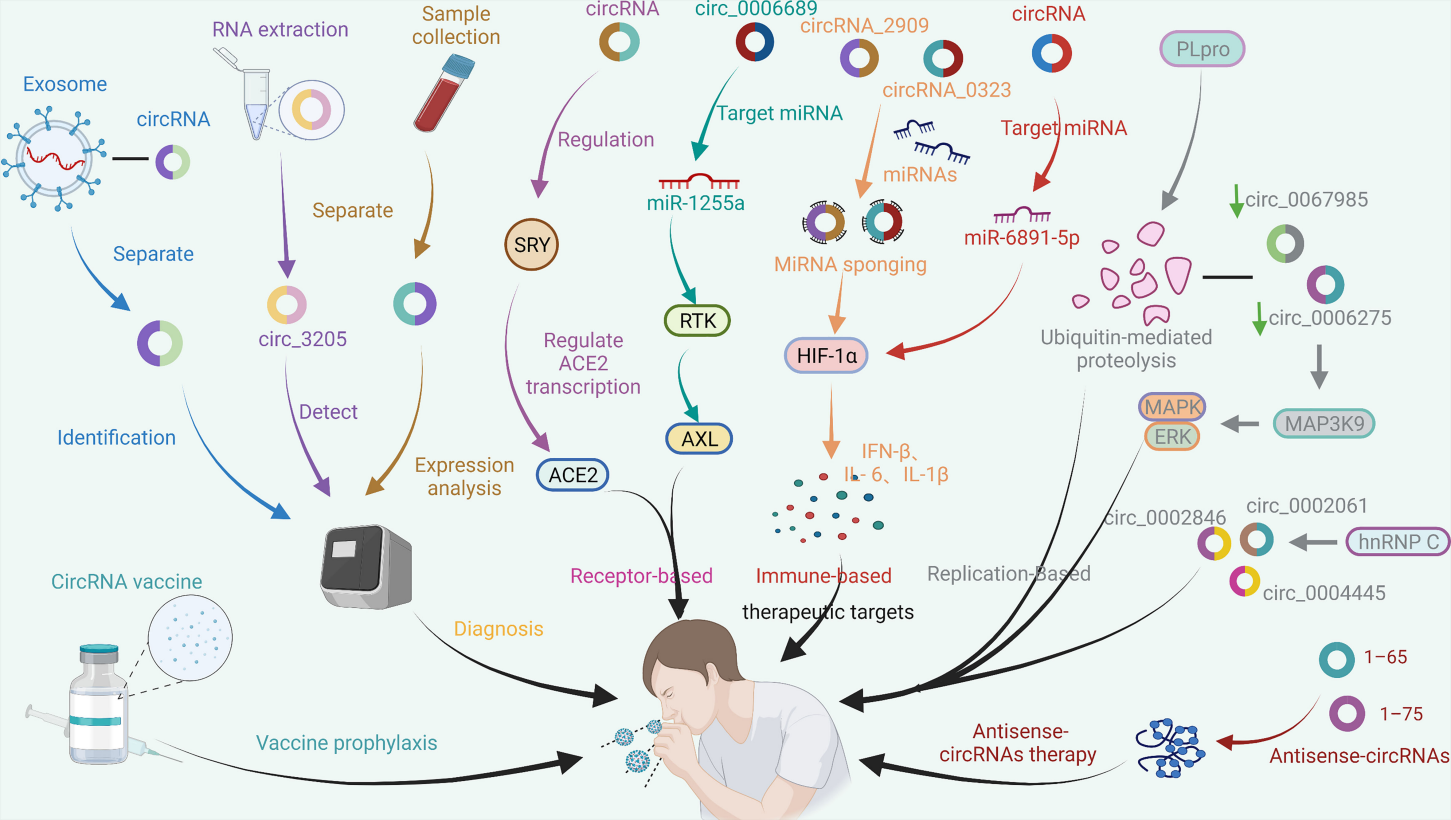
Potential application of circRNAs in the treatment of COVID-19. CircRNAs are a new class of regulatory factors that mediate host–virus interactions. The identification and isolation of exosome-associated circRNAs, virus-encoded circRNAs, and significantly DE circRNAs after SARS-CoV-2 infection may be helpful in the diagnosis of COVID-19. In addition, circRNAs may serve as potential therapeutic targets against COVID-19 by indirectly regulating the expression of host receptors, such as ACE2 and AXL, that bind to SARS-CoV-2; HIF-1α and other signalling pathways related to the immune response; and multiple signalling pathways related to SARS-CoV-2 replication. Additionally, vaccines based on circRNAs and antisense circRNAs have shown initial effectiveness in preventing and inhibiting SARS-CoV-2. The coloured arrows in the figure are only for the convenience of differentiation and have no special meaning.

By far, the new mutated strain of SARS-CoV-2, Omicron, has once again caused a global panic. At this stage, vaccination is the most promising way to end the COVID-19 pandemic. However, the efficacy of vaccines and the onset of adverse reactions vary among individuals. Information on COVID-19 vaccines that have been certified for emergency use by the WHO and are in the process of being evaluated is summarized in **Table 1** (132–149). Traditional vaccines and nucleic acid vaccines against SARS-CoV-2 have been extensively developed. Among them, an mRNA vaccine was the first to be officially approved by the FDA for use in the COVID-19 pandemic due to its advantages of rapid production, low cost, and rapid response to SARS-CoV-2 infection. However, in the face of rapidly mutating virus strains, alternative vaccines with high efficacy, high design flexibility, and fast production have not yet been developed. With the development of RNA vaccines, improving RNA stability has become a huge challenge. Fortunately, prior to this challenge, circRNAs showed great potential. Unlike linear mRNA, circRNA is highly stable and has a long half-life because its covalently closed-loop structure protects it from exonuclease-mediated degradation (150–152). However, only a few endogenous circRNAs have been demonstrated to serve as templates for protein translation, but several studies have shown that m6A modifications introduced into the ribosomal entry site (IRES) or 5’-untranslated region *via* artificial engineering can promote the extensive translation of circRNAs (110, 153–155). Recently, Liang Qu et al. (10) rapidly synthesized a highly stable circRNA-RBD vaccine (a circRNA vaccine that encodes the receptor-binding domain (RBD) of the SARS-CoV-2 S protein trimer) through *in vitro* transcription and found that the vaccine was capable of inducing potent and sustained anti-SARS-CoV-2-RBD neutralizing antibodies and Th1-biased T-cell responses in mice. Furthermore, the production of highly active neutralizing antibodies of the corresponding Beta variant was successfully induced in mice by using the circRNA vaccine encoding the RBD variant (K417N-E484K-501Y). Moreover, the latest research results have revealed that circRNA-RBD-Delta can elicit high levels of neutralizing antibodies against the Delta and Omicron variants compared to the circRNA-RBD-Omicron vaccine, which only induces effective neutralizing antibodies against Omicron. In addition, Seephetdee C et al. (20) found that SARS-CoV-2 circRNA vaccine VFLIP-X induces humoral and cellular immune responses that provide broad immune responses against emerging SARS-CoV-2 variants in mice. The results showed that circRNA vaccines can not only effectively prevent SARS-CoV-2 infection but can also quickly adapt to emerging SARS-CoV-2 mutant strains. Additionally, compared with the preparation method for conventional inactivated vaccines, which requires obtaining virus strains and then expanding and culturing live viruses, circRNA technology allows rapid development of new vaccines by only obtaining virus sequences or mutated sequences. CircRNA vaccines have the advantages of strong stability, immunogen coding ability, self-adjuvant, rapid mass production *in vitro*, and no needed nucleotide modification. CircRNAs can also be used to express nanobodies or ACE2 decoys to neutralize SARS-CoV-2 pseudovirus (10). Recently, Breuer J et al. (156) found that artificial circRNAs can bypass the cellular RNA sensors and that are not recognized by the innate immune system. Moreover, the antisense circRNAs 1–65 and 1–75 designed by Pfafenrot C et al. (14) significantly inhibited viral replication by specifically targeting specific 5’-UTR regions and sgRNAs of the SARS-CoV-2 genome. The potential applications of circRNAs for the diagnosis, treatment and prognosis of COVID-19 are shown in **Figure 5**, which indicates that circRNAs have very good application prospects in the fight against SARS-CoV-2 variant viruses, and circRNA vaccines and artificial circRNAs can be used as new vaccines and therapeutic platforms in the COVID-19 pandemic.

**Table 1:** Status of COVID-19 vaccines within WHO Emergency Use Listing (EUL)/Prequalification (PQ).

Vaccine	WHO EUL Holder	National Regulatory System (NRA) of record	Recommendation issued	Suitable age	Type of vaccine	Working principle	Disadvantages	Reference
COMIRNATY®	BioNTech Manufacturing GmbH	European Medicines Agency		aged 5 years and older	Nucleic acid vaccine	mRNA encoding SARS-CoV-2 spike protein	broken down shortly after vaccination myocarditis pericarditiserythema multiformeAllergic reactions	(132)
		Food and Drug Administration	16-Jul-21					
VAXZEVRIA	AstraZeneca AB / SK Bioscience Co. Ltd	European Medicines Agency	15-Feb-21	aged 18 years and older	Adenovirus vector vaccine	Adenovirus recombinantly containing a gene for the production of SARS-CoV-2 spike-in protein	Thrombosis in combination with thrombocytopenia(TTS),Guillain-Barré syndromeangioedema,capillary leak syndrome,Allergic reactions	(133)
		European Medicines Agency	15-Apr-21					
		Ministry of Health, Labour and Welfare	09-Jul-21					
	AstraZeneca AB	Therapeutic Goods Administration	09-Jul-21					
		Health Canada						
		COFEPRIS (DP) ANMAT (DS)						
COVISHIELD™	Serum Institute of India Pvt. Ltd	Central Drugs Standard Control Organization	15-Feb-21	aged 18 years and older	Adenovirus vector vaccine	Adenovirus recombinantly containing a gene for the production of SARS-CoV-2 spike-in protein	Thrombosis in combination with thrombocytopenia TTS),Allergic reactions	(134)
Ad26.COV2-S [recombinant]	Janssen–Cilag International NV	European Medicines Agency	12-Mar-21	aged 18 years and older	Adenovirus vector vaccine	encoding a full-length and stabilized SARS-CoV-2 spike protein	thrombosis with thrombocytopenia syndrome [TTS], capillary leak syndrome, and Guillain-Barré syndrome	(135)
SPIKEVAX	Moderna Biotech	European Medicines Agency	30-Apr-21	aged 12 years and older	Nucleic acid vaccine	mRNA encoding SARS-CoV-2 spike protein	broken down shortly after vaccination,erythema multiforme,Allergic reactions	(136)
	ModernaTX, Inc	Ministry of Food and Drug Safety (MFDS)						
		Food and Drug Administration						
Inactivated COVID-19 Vaccine (Vero Cell)	Beijing Institute of Biological Products Co., Ltd. (BIBP)	National Medicinal Products Association		18 to 59 years of age	Inactivated vaccine	The antibodies against the SARS-CoV-2 can be produced after vaccination	local injection site reactions	(137)
CoronaVac	Sinovac Life Sciences Co., Ltd	National Medical Products Administration	01-Jun-21	18 to 59 years of age	Inactivated vaccine	The antibodies against the SARS-CoV-2 can be produced after vaccination	local injection site reactions	(138)
COVAXIN®	Bharat Biotech International Ltd	Central Drugs Standard Control Organization	03-Nov-21	aged 18 years and older	Inactivated vaccine	The antibodies against the SARS-CoV-2 can be produced after vaccination	Headaches,Fever	(139)
COVOVAX™	Serum Institute of India Pvt. Ltd	Central Drugs Standard Control Organization		aged 18 years and older	Recombinant subunit vaccine	The antibodies against the SARS-CoV-2 can be produced after vaccination	local injection site reactions	(140)
NUVAXOVID™	Novavax CZ a.s.	European Medicines Agency		aged 18 years and older	Recombinant subunit vaccine	The antibodies against the SARS-CoV-2 can be produced after vaccination	local injection site reactions	(140)
Sputnik V	Russian Direct Investment Fund	Russian NRA	/	aged 18 years and older	Adenovirus vector vaccine	The antibodies against the SARS-CoV-2 can be produced after vaccination	Localised pain, weakness, headaches and joint pain	(141)
Inactivated SARS-CoV-2 Vaccine (Vero Cell)	Wuhan Institute of Biological Products Co Ltd	National Medicinal Products Association	/	18 to 59 years of age	Inactivated vaccine	The antibodies against the SARS-CoV-2 can be produced after vaccination	local injection site reactions	(142)
Ad5-nCoV	CanSinoBIO	National Medicinal Products Association	/	/	Recombinant subunit vaccine	Adenovirus recombinantly containing a gene for the production of SARS-CoV-2 spike-in protein	local injection site reactions,Headache, Drowsiness and Muscle aches	(143)
CoV2 preS dTM-AS03 vaccine	SANOFI	European Medicines Agency	/	/	Adjuvanted soluble protein vaccines	robust induction of antibody responses	local injection site reactions,Headaches,Fever	(144)
SCB-2019	Clover Biopharmaceuticals	National Medicinal Products Association	/	/	Recombinant subunit vaccine	The antibodies against the SARS-CoV-2 can be produced after vaccination	local injection site reactions	(145)
Recombinant Novel Coronavirus Vaccine (CHO Cell)	Zhifei Longcom, China	National Medicinal Products Association	/	/	Recombinant subunit vaccine	The antibodies against the SARS-CoV-2 can be produced after vaccination	local injection site reactions	(146)
Zorecimeran (INN) concentrate and solvent for dispersion for injection; Company code: CVnCoV/CV07050101	Curevac	European Medicines Agency	/	/	Nucleic acid vaccine	mRNA-based vaccine encapsulated in lipid nanoparticle (LNP)	Headache, fatigue, chills and pain at the injection site	(147)
EpiVacCorona	Vector State Research Centre of Viralogy and Biotechnology	Russian NRA	/	/	Adenovirus vector vaccine	The immune system is stimulated to neutralize the virus by peptides-short fragments of viral protein	/	(148)
SARS-CoV-2 Vaccine, Inactivated (Vero Cell)	IMBCAMS, China	National Medicinal Products Association	/	18 to 59 years of age	Inactivated vaccine	The antibodies against the SARS-CoV-2 can be produced after vaccination	Redness and swelling at the injection site, induration and fever at the injection site	(142)
Soberana 01, Soberana 02 Soberana Plus Abdala	BioCubaFarma - Cuba	CECMED	/	/	Conjugate protein vaccines	SARS-CoV-2 spike protein conjugated chemically to meningococcal B or tetanus toxoid or Aluminum	/	(149)

In general, circRNAs are highly resistant to RNAse R due to their unique ring structure and are more conservative and stable than lncRNAs and miRNAs. CircRNAs can exist stably in cells or tissues and have become the star molecules in the field of ncRNA. Furthermore, circRNAs have the potential to be molecular markers of viral infectious diseases, which can provide a scientific basis for early diagnosis of diseases and the search for potential therapeutic targets. Taking circRNAs as an entry point to study the interaction between viral infection and the host will help clarify the function of circRNAs and thus the pathogenic mechanism of coronaviruses. Therefore, circRNAs may prove to be helpful as diagnostic markers and therapeutic agents against COVID-19.

## Author contributions

XG, DF, YL and ML conceived the idea, analysis of literature, and writing of the manuscript; XG, XD and NC collected and read the literature and revised the article; MZ and ML read through and corrected the manuscript. All authors contributed to the article and approved the submitted version.

## Funding

This work was supported by the National Natural Science Foundation of China [grant numbers 81800434], Grant of Sichuan Province Science and Technology Agency Grant [2019YJ0487].

## Acknowledgments

Figures were created with ©BioRender - biorender.com.

## Conflict of interest

The authors declare that the research was conducted in the absence of any commercial or financial relationships that could be construed as a potential conflict of interest.

## Publisher’s note

All claims expressed in this article are solely those of the authors and do not necessarily represent those of their affiliated organizations, or those of the publisher, the editors and the reviewers. Any product that may be evaluated in this article, or claim that may be made by its manufacturer, is not guaranteed or endorsed by the publisher.

## Glossary

**Table:**

COVID-19	coronavirus disease 2019
RNAse R	ribonuclease R
SARS-CoV-2	severe acute respiratory syndrome coronavirus 2
CoVs	coronaviruses
PKR	Protein Kinase R
RNase L	ribonuclease L
NF90	nuclear factor 90
NF110	nuclear factor 110
Calu-3	human lung adenocarcinoma cells
SARS-CoV-1	severe acute respiratory syndrome coronavirus 1
MERS-CoV	middle east respiratory syndrome coronavirus
ORF	open reading frame
nsps	nonstructural proteins
S	spike
E	envelope
M	membrane
N	nucleocapsid
ACE2	angiotensin-converting enzyme 2
DPP4	dipeptidyl peptidase 4
TMPRSS2	transmembrane serine protease 2
CatB	cathepsin B
CatL	cathepsin L
AAK1	AP2-associated kinase 1
TLRs	toll-like receptors
TF	transcription factor
SIRs	systemic inflammatory responses
IL-6	interleukin-6
ncRNAs	non-coding RNAs
hpi	hours post-infection
ecircRNA	exonic circRNA
ciRNA	intronic circRNA
EIciRNA	exon-intronic circRNA
ceRNA	competing endogenouse RNA
miRNA	microRNA
lncRNA	long noncoding RNA
mRNA	messenger RNA
HFL-I	human embryonic lung fibroblast cell
ILF3	human interleukin-enhanced binding factor 3
dsRNA	double-stranded RNA
CircRNPs	circRNA-protein complexes
RIG-I	retinoic acid inducible gene-I
HIF-1	hypoxia-inducible factor-1
mTOR	mammalian leukemia target of pamycin
TNF	tumor necrosis factor
MAPK	mitogen-activated protein kinase
ISG	interferon-stimulated gene
IFN	interferon
OAS1-3	2'-5' oligoadenylate synthase
Ifit1-3	interferon-inducible protein
Th1	T helper cell type 1
2'-5'A	2'-5' oligoadenylate
PKR	Protein Kinase R
RA	arthritic
HUVSMCs	human vascular smooth muscle cells
HUVECs	human vascular endothelial cells
RLRs	RIG-I-like receptors
FSJs	forward splicing junctions
SRY	sex-determining region Y
AXL	AXL receptor tyrosine kinase
RTK	receptor tyrosine kinase
NF-kB	nuclear factor-kB
NOS2	inducible nitric oxide synthase
PLpro	papain-like protease
ERK	extracellular signal-regulated kinase
RBD	receptor-binding domain
